# Genome-wide association mapping for milk fat composition and fine mapping of a QTL for de novo synthesis of milk fatty acids on bovine chromosome 13

**DOI:** 10.1186/s12711-017-0294-5

**Published:** 2017-02-13

**Authors:** Hanne Gro Olsen, Tim Martin Knutsen, Achim Kohler, Morten Svendsen, Lars Gidskehaug, Harald Grove, Torfinn Nome, Marte Sodeland, Kristil Kindem Sundsaasen, Matthew Peter Kent, Harald Martens, Sigbjørn Lien

**Affiliations:** 1 Centre for Integrative Genetics (CIGENE), Department of Animal and Aquacultural Sciences, Norwegian University of Life Sciences, PO Box 5003, 1432 Ås, Norway; 2 Department of Mathematical Sciences and Technology, Norwegian University of Life Sciences, PO Box 5003, 1432 Ås, Norway; 3 Centre for Biospectroscopy and Data Modeling, Nofima AS, Osloveien 1, 1430 Ås, Norway; 4 Geno Breeding and AI Association, 1432 Ås, Norway; 5 CAMO Software AS, Nedre Vollgate 8, 0158 Oslo, Norway; 6 Institute of Marine Research, Flødevigen, 4817 His, Norway; 7 Department of Natural Sciences, Faculty of Engineering and Science, University of Agder, PO Box 422, 4604 Kristiansand, Norway; 8 Department of Engineering Cybernetics, Norwegian University of Science and Technology, 7034 Trondheim, Norway

## Abstract

**Background:**

Bovine milk is widely regarded as a nutritious food source for humans, although the effects of individual fatty acids on human health is a subject of debate. Based on the assumption that genomic selection offers potential to improve milk fat composition, there is strong interest to understand more about the genetic factors that influence the biosynthesis of bovine milk and the molecular mechanisms that regulate milk fat synthesis and secretion. For this reason, the work reported here aimed at identifying genetic variants that affect milk fatty acid composition in Norwegian Red cattle. Milk fatty acid composition was predicted from the nation-wide recording scheme using Fourier transform infrared spectroscopy data and applied to estimate heritabilities for 36 individual and combined fatty acid traits. The recordings were used to generate daughter yield deviations that were first applied in a genome-wide association (GWAS) study with 17,343 markers to identify quantitative trait loci (QTL) affecting fatty acid composition, and next on high-density and sequence-level datasets to fine-map the most significant QTL on BTA13 (BTA for *Bos taurus* chromosome).

**Results:**

The initial GWAS revealed 200 significant associations, with the strongest signals on BTA1, 13 and 15. The BTA13 QTL highlighted a strong functional candidate gene for de novo synthesis of short- and medium-chained saturated fatty acids; *acyl*-*CoA synthetase short*-*chain family member 2*. However, subsequent fine-mapping using single nucleotide polymorphisms (SNPs) from a high-density chip and variants detected by resequencing showed that the effect was more likely caused by a second nearby gene; *nuclear receptor coactivator 6* (*NCOA6*). These findings were confirmed with results from haplotype studies. NCOA6 is a nuclear receptor that interacts with transcription factors such as PPARγ, which is a major regulator of bovine milk fat synthesis.

**Conclusions:**

An initial GWAS revealed a highly significant QTL for de novo-synthesized fatty acids on BTA13 and was followed by fine-mapping of the QTL within *NCOA6*. The most significant SNPs were either synonymous or situated in introns; more research is needed to uncover the underlying causal DNA variation(s).

**Electronic supplementary material:**

The online version of this article (doi:10.1186/s12711-017-0294-5) contains supplementary material, which is available to authorized users.

## Background

While bovine milk is generally regarded as being highly nutritious for humans and serving as an important source of proteins, fat, minerals, vitamins and bio-active lipid components, the net effect of dairy fat on human health is strongly debated. This is because saturated fatty acids (FA), which constitute roughly 60 to 70% of the FA in milk, have been associated with cardiovascular disease and obesity, while mono-and polyunsaturated FA have been associated with positive effects on both cardiovascular health and diabetes (see e.g., [1] for a review).

Biosynthesis of bovine milk fat is a complex process, which is regulated by a network of genes that encode a set of transcription regulators and nuclear factors [2]. In essence, milk FA are derived via one of two major pathways: either by de novo synthesis in the mammary gland, or by direct transport from rumen to mammary gland through blood. More specifically, short- and medium-chained saturated FA C4:0–C14:0, as well as approximately 50% of C16:0, are synthesized de novo in the mammary gland from C2 and C4 precursors. The remaining C16:0 and long-chained saturated FA are derived from circulating plasma lipids which originate from the diet or from lipolysis of adipose tissue triacylglycerols. Long-chained FA are mainly saturated in the rumen. Both the long- and the medium-chained acids can be desaturated by Δ^9^-desaturase to their *cis*-*9* monounsaturated counterparts.

Milk FA composition varies among individuals, as well as within individuals depending on their lactation stage [3, 4]. It is highly affected by environmental factors such as feeding, udder health and season, but is also genetically influenced. Substantial genetic variation associated with bovine milk fat composition has been reported [5–10], with estimated heritabilities for individual FA being low to moderate (usually in the range from 0.05 to 0.40). This raises the possibility to improve nutritional properties of milk fat by selective breeding.

Traditionally, detailed milk fat composition is determined by gas chromatography (GC) analysis. This is an accurate but expensive method and is not suitable for routine milk recording. Recent studies showed that Fourier transform infrared spectroscopy (FTIR) data, calibrated against gas chromatography with flame ionization detector (GC–FID) reference data from the same samples, has the potential to provide detailed prediction of milk fat composition [11–19]. An advantage of this approach is that the millions of records obtained by routine recording of cows can be used to estimate genetic parameters and improve traits by breeding. In this study, we used such data to perform a genome-wide association analysis (GWAS) in Norwegian Red cattle to search for genes that affect milk fat composition. A candidate region on BTA13 (BTA for *Bos taurus* chromosome) that influences de novo synthesis of short- and medium-chained FA was fine-mapped and re-analyzed for novel single nucleotide polymorphisms (SNPs) that were detected by re-sequencing in order to attempt to identify the underlying causal DNA variation.

## Methods

### Estimation of bovine milk fat composition from FTIR spectroscopy data

To obtain a calibration model for FTIR spectra, 262 milk samples obtained from a feeding experiment [14] and 616 samples from field sampling were analyzed in parallel by FTIR spectroscopy and GC–FID reference analysis. All samples were from Norwegian Red (NR) cows. FTIR analyses were performed using an FT-IR MilkoScan Combifoss 6500 instrument (Foss, Hillerød, Denmark). Samples were homogenized and temperature-regulated before entering a cuvette (37 μm) for transmission measurements in the spectral range from 925 to 5011 cm^−1^. The instrument was equipped with a DTGS detector. All spectra were transformed from transmittance to absorbance units. Absorbance spectra were preprocessed by taking the second derivative using Savitzky–Golay algorithm with a polynomial of degree 2 and a window size of 9 channels followed by extended multiplicative signal correction [20] in order to correct for baseline variations and multiplicative effect [21]. FTIR spectra (regressors) were subsequently calibrated against GC–FID reference values (regressands) by using powered partial least squares regression (PPLSR, [22]). Regressands were presented as percentages of GC–FID fatty acid values to total fat in order to reduce to a minimum value the correlation between the FA and total fat in milk samples. Calibration was assessed by 20-fold cross-validation, i.e. the calibration data was divided randomly into 20 segments and each of them was used as an independent test set at a time. The number of components was selected automatically by evaluating if the improvement of the cross-validated prediction of the regressands was significant when the number of PLS components (linear channel combinations) increased in the reduced-rank PPLSR model. If improvement of the calibration model was not significant when moving from component number *A* to component number *A* + 1, *A* was chosen as the optimal number of components. However, in order to avoid overfitting, the maximum number of components was set to 25.

The traits that were calibrated in this study included 24 individual FA and 12 combined traits. Individual FA included seven short- and medium-chained, even-numbered saturated FA (C4:0, C6:0, C8:0, C10:0, C12:0, C14:0, C16:0), two long-chained saturated FA (C18:0, C20:0), two odd-numbered saturated FA (C15:0, C17:0), seven monounsaturated FA (C14:1*cis*-9, C16:1*cis*-9, C18:1*cis*-9, C18:1*cis*-11, C18:1*trans*-9, C18:1*trans*-10, C18:1*trans*-11) and six polyunsaturated FA [C18:2*cis*-9,*cis*-12, C18:3*cis*-9,*cis*-12,*cis*-15, arachinonic acid (ARA), conjugated linoleic acid (CLA), docosahexaenoic acid (DHA) and eicosapentaenoic acid (EPA)]. The combined traits were CIS (% of FA with *cis* bonds), TRANS (% of FA with *trans* bonds), TRANS:CIS (*trans*:*cis* ratio), N3 (total amount of omega-3 FA), N6 (total amount of omega-6 FA), N3:N6 (omega-3:omega-6 ratio), DNS (de novo FA synthesis, i.e., sum of the short-chained FA C6:0–C12:0), SAT (% of saturated FA), MUFA (% monounsaturated FA), PUFA (% polyunsaturated FA), TOTAL (total fat yield), and iodine value. NEFA (free FA) and UREA were also included in the GWAS, but these traits have built-in prediction equations in the FT-IR instrument and are stored as a routine procedure in the Norwegian Dairy Herd recording system as parameters of milk quality and feeding, and were therefore not calibrated in this study.

### Estimation of variance components and daughter yield deviations

The obtained calibration models were applied to about 1,650,000 infrared spectra from the Regional Laboratories of the Norwegian Herd recording system for the periods February to November 2007 and July 2008 to March 2009 (spectra from November 2007 to July 2008 were missing due to technical problems with the storage of data during that period). Predicted values of bimonthly test day samples were used for further statistical analyses. The ~1,650,000 FTIR-based FA profile predictions for individual cows (Y) were related to the pedigree structure of the NR population. To condense the information for genetic analyses, only a subset of the data was used. The cows had to be in 1st to 4th lactation and the test-days between 10 and 320 days after calving. The milk yield at the test-day had to be between 5 and 50 kg, and the fat percentage between 1.75 and 7.0. These criteria were designed to remove obvious outliers. Finally, the sire had to be an artificial insemination (AI) NR bull. Milk samples were recorded on a bimonthly basis. This left 950,170 profiles from 300,126 cows that were daughters from 1095 sires, with a total number of animals in the pedigree of 871,455 animals.

The data were analyzed with the following mixed linear animal repeatability model:

$${\text{Y}} = {\text{RYM}}_{\text{i}} + {\text{RPL}}_{\text{j}} + {\text{htd}}_{\text{k}} + {\text{pe}}_{\text{l}} + {\text{a}}_{\text{m}} + {\text{e}}_{\text{ijklm}} ,$$

where RYM is the fixed effect of region (9 regions) by year and month of the test-day, with i ranging from 1 to 170; RPL is the fixed effect of region by lactation number by 10-day period in lactation of the test-day, with j ranging from 1 to 1116; htd is the random effect of herd by test-day, with k ranging from 1 to 83,850; pe is the random permanent environmental effect of the cow on her repeated records, with l ranging from 1 to 300,126; a is a random additive genetic effect of the animal, with m ranging from 1 to 871,455; and e is a random residual effect.

The distributional assumptions for the random effects were the following: htd ~ *N*(**0**, **I** $\upsigma_{\text{htd}}^{2}$), pe ~ *N*(**0**, **I** $\upsigma_{\text{pe}}^{2}$), a ~ *N*(**0**, **A** $\upsigma_{\text{a}}^{2}$), and e ~ *N*(**0**, **I** $\upsigma_{\text{e}}^{2}$), where **0** is a null vector, **I** an identity matrix and **A** is the additive genetic relationship matrix.

The variance components were estimated by using the DMU software [23] and an average information algorithm. Given the variance components, breeding values and fixed effects were estimated by the DMU software using an iteration on data algorithm.

Daughter yield deviations (DYD) for the GWAS were then derived from these results as the sire averages of daughters’ predicted FA compositions, which were each corrected for her fixed effects, non-genetic random effects and half of her dam’s genetic effect. The number of bulls with DYD and genotype information varied from step to step as described below, mainly because genotyping on the SNP chips (see below) was performed on animals with trait data for many of the traits in the breeding goal, and was not specific to animals with DYD for the milk FA. The average number of daughters per bull was ~300 in all steps.

### Genotypes for genome-wide association analyses

Initial genotyping for the GWAS was performed on 2552 NR AI bulls using the Affymetrix 25K bovine SNP chip (Affymetrix, Santa Clara, CA, USA) as described in [24]. SNP filtering reduced the number of useful SNPs to 17,343 (see [24] for details). SNPs were positioned on the genome by using the UMD 3.1 assembly [25]. DYD were available for 991 of the 2552 bulls.

### Construction of a high-density SNP dataset with 16,567 SNPs on BTA13

A dense SNP map for fine-mapping on BTA13 was constructed by combining genotypes from the Affymetrix 25K SNP chip with genotypes from Illumina’s BovineSNP50 (54K) and BovineHD (777K) BeadChips (Illumina, San Diego, CA, USA). A total of 1575 NR bulls were genotyped with the 54K chip, 536 of these bulls were also among the 2552 animals genotyped with the 25K chip. Next, 384 of the 1575 bulls were genotyped with the 777K chip. The three datasets were filtered to remove SNPs with a minor allele frequency lower than 0.05 and all remaining SNPs were positioned according to the UMD 3.1 assembly. The 25K dataset was imputed to 54K before the combined 54K dataset was imputed to 777K. All imputations and phasing were performed using BEAGLE v3.3.1 [26] with default options. Phase information of the imputed haplotypes was used to identify double recombinants and if possible correct or remove these. The resulting dataset consisted of 3289 NR bulls and 16,567 SNPs on BTA13. DYD were available for 1024 of the bulls, with an average of 278 daughters per son. The 991 bulls used in the previous GWAS step were among these 1024 bulls.

### Genome re-sequencing and construction of a sequence-level SNP dataset for the candidate gene region

Whole-genome re-sequencing data were obtained for five NR elite bulls on an Illumina Genome Analyzer GAIIx instrument (Illumina, San Diego, CA, USA) with 2× 108 paired end reads. The five bulls were selected based on their large numbers of offspring and minimum relationships and therefore represented the genetic diversity of the population. Library preparation was performed using a TruSeq SBS V2-GA kit (Illumina, San Diego, CA, USA). Adaptor- and quality-trimming of raw reads in FASTQ-format was performed using the FASTX-toolkit v0.0.13 [27]. The reads were aligned against BTA13 (bovine reference genome assembly UMD 3.1) using Bowtie v0.12.7 [28] with default parameters. Sorting, marking of PCR duplicates and indexing of the resulting SAM files were performed using Samtools v0.1.17 [29]. Between 98.7 and 99.7% of the reads were mapped to the bovine reference genome assembly UMD 3.1, including all chromosomes and unplaced scaffolds. The average whole-genome sequence coverage for each animal was estimated by dividing the total number of sequenced fragments times read length by the length of the bovine genome (3 gigabases). Two bulls in the dataset had an average whole-genome sequence coverage of about 10×, while three bulls had an average coverage of 4×. Variant calling was performed with Freebayes v0.1.0 [30] with a minimum read coverage of 2 and a minimum alternate allele count of 1. The settings were chosen to maximize calling sensitivity given the relatively low sequence coverage for three of the samples.

Since the parameters for variant calling were set to detect as much variation as possible, rather strict criteria for selecting a novel SNP for further genotyping were set. A total of 1260 SNPs were found within the two genes *nuclear receptor coactivator 6* (*NCOA6*) and *acyl*-*CoA synthetase short*-*chain family member 2* (*ACSS2*) or within 2000 bp on either side of these genes. Among these 1260 SNPs, all SNPs in exons and UTR were selected for genotyping together with intronic SNPs that were present in the dbSNP database [31] and co-segregated with the most significant SNPs from the analyses of the high-density data on BTA13. This approach resulted in 71 SNPs that were used to genotype 570 animals. However, as expected given the relatively relaxed SNP detection criteria applied initially, several of these SNPs were found to be monomorphic and hence to be false positives after genotyping. Only 17 SNPs passed all the steps. Of these, two exonic and 11 intronic SNPs were positioned within *NCOA6*, one exonic and two intronic SNPs were located within *ACSS2*, and one SNP was found in the neighboring gene *GSS*. In order to include missing genotypes, to include bulls with trait data that were not genotyped, and to also cover the regions outside the two genes, the 17 novel SNPs together with SNPs from the BovineHD array positioned in the QTL region were imputed by using BEAGLE v3.3.1 [26]. Hence, the final map consisted of 204 SNPs that were located between 63,488,876 and 65,786,868 bp. Of these, 15 and 9 SNPs were located within *NCOA6* and *ACSS2*, respectively. The total number of bulls with genotypes for the 204 SNPs and trait data in the dataset was equal to 782, and the average number of daughters per bull was equal to 362. This dataset was used to fine-map the candidate gene region and for haplotype analyses. Names, positions and primer sequences for the 17 novel SNPs detected by re-sequencing are in Additional file 1: Table S1.

### Single-marker association studies

A single-marker association model was used for the GWAS, the re-sequenced BTA13 map and the candidate gene map. The model that was fitted to the performance data for each trait and each SNP was as follows:

$${\text{DYD}}_{\text{i}} =\upmu + {\text{m}} + {\text{a}}_{\text{i}} + {\text{e}}_{\text{i}} ,$$

where DYD_i_ is performance of bull i, μ is the overall mean, m is a random SNP effect, a_i_ is a random polygenic effect of bull i, and e_i_ is a residual effect. We used a random SNP effect because since we performed a REML likelihood ratio test using REML, it was necessary to have the same fixed effects in H1 and H0 (i.e., the model with and without the SNP effect) for the two models to be comparable. Alleles were coded as numbers from 1 to 4 (i.e., A = 1, C = 2, G = 3 and T = 4). A random polygenic effect was included to account for putative genetic differences among bulls other than the SNP effect. The DYD were weighed by the number of daughters. The variances were estimated from the data. The SNP effect m was assumed to follow a normal distribution ~*N*(**0**, $\upsigma_{\text{m}}^{2}$), where $\upsigma_{\text{m}}^{2}$ is the SNP variance. The polygenic effect a was assumed to follow a normal distribution ~*N*(**0**, **A** $\upsigma_{\text{a}}^{2}$), where **A** is the relationship matrix among the analyzed bulls derived from the pedigree, and $\upsigma_{\text{a}}^{2}$ is the additive genetic variance. The residual effect e was assumed to follow a normal distribution ~*N*(**0**, **W** $\upsigma_{\text{e}}^{2}$), where $\upsigma_{\text{e}}^{2}$ is the environmental variance and **W** is the matrix of weights computed by ASReml based on the number of daughters in the DYD mean.

Significance levels for the random SNP effects were obtained from the log-likelihoods (logL) of a model that includes the SNP effect [LogL(H1)] as well as those of a model without this SNP effect [LogL(H0)], which were both calculated for each SNP using the ASREML package version 2.0 [32]. A likelihood ratio test-statistic (LRT) was calculated as LRT = 2 * [LogL(H1) − LogL(H0)]. Following Baret et al. [33], the distribution of the LRT under the null hypothesis can be seen as a mixture of two Chi square distributions with 0 and 1 degree of freedom, respectively. The significance levels are then obtained from a Chi square distribution with 1 degree of freedom but doubling the probability levels. Due to the amount of multiple-testing performed, we required a rather stringent significance threshold of p = 0.00025. Thus, the corresponding LRT were obtained from a Chi square distribution with 1 degree of freedom and p = 0.0005, and must be equal to 12.12 or more.

### Correction for the most significant QTL

In order to determine if more than one QTL was segregating in the candidate region, the effect of the most significant SNP from the single-marker analyses of the candidate gene region was corrected for by including it as a fixed effect in the single-marker model and repeating the analysis for all other SNPs in the candidate gene region.

### Haplotype analyses

Pair-wise LD measure (r^2^) was estimated for all SNP pairs in the candidate gene region on BTA13 using Haploview 4.2 [34]. Haploptype blocks were defined manually. Block 1 was a narrow *NCOA6* block that contained the most significant SNPs (SNPs 98–102), block 2 was a wider *NCOA6* block (SNPs 98–108), block 3 spanned *ACSS2* (SNPs 114–122), while block 4 included SNPs that were present in both *NCOA6* and *ACSS2* (SNPs 98–125). For each of the defined blocks, haplotypes for each sire were determined from the phased genotypes. Since very few sires were homozygous for the least frequent haplotypes, sires with one or two copies of the haplotype were grouped and a two-sample *t* test was performed in R [35] to test for differences in mean phenotypic value between this group and the remaining sires.

## Results and discussion

### FTIR spectroscopy and variance component estimation

A key requirement of this study was to be able to estimate FA composition in milk samples based on FTIR spectroscopy data using a GC–FID reference analysis method [14]. The results showed that 29 of the FA, together representing more than 90% of the total fat content, achieved cross-validated squared Pearson product-moment correlation coefficients (R^2^CV) above 0.5; these FA were therefore considered predictable and included in the further analyses. As shown in Table 1 and Additional file 2: Table S2, the highest concentrations of individual FA were found for C16:0, C18:1*cis*-9, C18:0 and C14:0 (mean concentrations equal to 25.25, 21.4, 11.29 and 11.21% of total fat, respectively). The best combined trait predictions were obtained for SAT, CIS and MUFA (R^2^CV = 0.96), while the best predictions for individual FA were found for C18:1*cis*-9 (R^2^CV = 0.94) and for C8:0 to C12:0 (R^2^CV = 0.91). The results showed that most major FA were predicted rather accurately, however with lower R^2^CV for C16:0, C14:0 and C18:0 (R^2^CV = 0.77, 0.73 and 0.54, respectively). The ability to predict a FA with high confidence depended strongly on its concentration, and FA with concentrations less than 1% generally showed low R^2^CV and were considered unpredictable (Table 1). There were exceptions to this with a few low-frequency FA that achieved high R^2^CV, which is most likely due to cross-correlation with more frequent, predictable FA. Correlations between predicted FA and total fat percentage were low to moderate (Table 1) and showed a general trend for negative correlations for longer unsaturated FA, and positive correlations for shorter saturated FA. Mean concentrations of each trait from the GC–FID reference analyses, R^2^CV, correlation coefficients between each predicted FA and total fat percentage as well as heritabilities are in Table 1, while all the results for the PPLSR calibration and the GC–FID reference values and variance components are in Additional file 2: Table S2.

**Table 1:** Mean concentrations, cross-validated squared correlation coefficients, correlations to total fat, and heritabilities for all calibrated traits

Trait	Cons	R^2^CV	Corr (SE)	h^2^ (SE)
C4:0	4.16	0.73	0.111 (0.039)	0.353 (0.004)
C6:0	2.48	0.89	0.104 (0.039)	0.231 (0.003)
C8:0	1.48	0.91	0.040 (0.039)	0.187 (0.003)
C10:0	3.2	0.91	0.034 (0.039)	0.171 (0.003)
C12:0	3.55	0.91	0.045 (0.039)	0.179 (0.003)
C14:0	11.21	0.86	0.077 (0.039)	0.109 (0.003)
C14:1*cis*-9	0.98	0.52	0.089 (0.039)	0.222 (0.003)
C15:0	1.0	0.59	0.071 (0.039)	0.146 (0.003)
C16:0	25.25	0.77	0.433 (0.035)	0.145 (0.003)
C16:1*cis*-9	1.17	0.51	0.392 (0.036)	0.146 (0.003)
C17:0	0.49	0.43	0.146 (0.039)	0.142 (0.003)
C18:0	11.29	0.54	−0.279 (0.038)	0.175 (0.003)
C18:1*trans*-9	0.24	0.74	−0.521 (0.033)	0.141 (0.002)
C18:1*trans*-10	0.36	0.56	−0.543 (0.033)	0.171 (0.003)
C18:1*trans*-11	1.33	0.67	−0.318 (0.037)	0.092 (0.002)
C18:1*cis*-9	21.4	0.94	−0.186 (0.038)	0.127 (0.003)
C18:1*cis*-11	0.79	0.73	−0.357 (0.037)	0.146 (0.003)
C18:2*cis*-9,*cis*-12	1.39	0.61	−0.409 (0.036)	0.172 (0.003)
C18:2*cis*-9,*trans*-11	0.62	0.65	−0.325 (0.037)	0.120 (0.002)
C18:3*cis*-9,*cis*-12,*cis*-15	0.54	0.42	−0.231 (0.038)	0.190 (0.003)
C20:0	0.2	0.39	−0.336 (0.037)	0.161 (0.003)
ARA	0.07	0.46	−0.052 (0.039)	0.236 (0.004)
EPA	0.06	0.16	0.088 (0.039)	0.173 (0.003)
DHA	0.02	0.62	−0.014 (0.039)	0.159 (0.003)
SAT	64.31	0.96	0.308 (0.037)	0.137 (0.003)
MUFA	26.28	0.96	−0.229 (0.038)	0.130 (0.003)
PUFA	2.7	0.72	−0.491 (0.034)	0.171 (0.003)
Iodine value	25.51	0.95	−0.241 (0.038)	0.144 (0.003)
CIS	26.43	0.96	−0.198 (0.038)	0.138 (0.003)
TRANS	2.56	0.73	−0.419 (0.036)	0.103 (0.002)
TRANS:CIS	0.1	0.64	−0.377 (0.036)	0.096 (0.002)
DNS	10.72	0.92	0.048 (0.039)	0.165 (0.003)
N3	0.62	0.37	−0.211 (0.038)	0.191 (0.003)
N6	1.47	0.62	−0.386 (0.036)	0.170 (0.003)
N3:N6	0.44	0.42	0.143 (0.039)	0.193 (0.003)
Total	93.29	0.59	0.377 (0.036)	0.106 (0.002)

Mean concentration from the GC–FID reference analyses (Cons), cross-validated squared Pearson product-moment correlation coefficients (R^2^CV), Pearson correlation coefficients between the predicted fatty acids and total fat percentage (corr) and standard errors of the correlation, heritabilities (h^2^) and standard errors of the heritability for all calibrated traits. The concentration is expressed as percentage by weight of total fatty acid content (on a fatty acid methyl ester basis), except iodine value, which is expressed as g I_2_/100 g of total fatty acid content

Several studies investigated the effectiveness of mid-infrared spectroscopy to predict bovine FA composition [11–19], and reported that accuracies vary due to differences in the number of samples, breeds, spectra pretreatments, reference methods and units of measure. In agreement with our findings, prediction accuracies are generally best for FA with high concentrations and for the short and medium-chained FA, C18:1*cis*-9, and for SAT and MUFA. Prediction accuracies were in general better when FA concentrations were expressed as a quantity per unit of milk rather than a quantity of total milk fat, which is most likely because FA concentrations are correlated to total fat, and predicting FA in milk on the basis of FTIR is the combined effect of predicting fat content and fat composition [11, 13, 16]. However, these correlations should be lower when FA concentrations are expressed as quantity of total milk fat when models are developed on the basis of fat as in our study. Soyeurt et al. [11] suggested that the predicted concentrations were not due to real absorbance values specific to FA if the calibration correlations were not higher than the correlations between total fat and FA. As shown in Additional file 2: Table S2, the squared correlations between a FA and total fat percentage were markedly lower than the R^2^CV for all FA and combined traits groups in our study, which indicated that the predicted concentrations are due to real absorbance values of the FA rather than to correlations to total fat only. Moreover, prediction accuracies for C8:0, C10:0, C12:0, C18:1*cis*-9, SAT and MUFA were as high as those reported with milk-based models [13, 15, 17–19]. C4:0 and C14:0 were predicted with somewhat poorer accuracies than those usually obtained with milk-based models, but with better accuracies than those obtained with fat-based models [11, 13, 19]. Predictions of C16:0 were comparable to those obtained with fat-based models [11, 13, 19].

In general, the selected number of components was large, but since the PPLSR model is very selective for each component, a larger number of selected components is expected than with a conventional PLSR model. In addition, the complexity of the calibration reference data used in this study was considerably higher and the level of variation of the data was much higher compared to those for the data reported in Afseth et al. [14], and thus the model is expected to be more complex. Compared to the reference data used in Afseth et al. [14], the current calibration set contains many samples with a considerable higher proportion of unsaturated acids.

Relatively high heritabilities were estimated from the FTIR predictions (Table 1). Estimates for the predictable FA ranged from 0.09 for C18:1*trans*-11 to 0.35 for C4:0. Short and medium length FA were slightly more heritable than longer and unsaturated FA. This is as expected since the shorter saturated FA are mainly synthesized by the animal, while longer unsaturated FA originate predominately from the diet. The heritability for the sum of polyunsaturated FA (PUFA) was somewhat higher than that for the sum of monounsaturated (MUFA) and saturated (SAT) FA (h^2^ = 0.171, 0.130 and 0.137, respectively). These results can be explained by the fact that all three indices (SAT, MUFA and PUFA) reflect a combination of genetic and environmental factors, and that the prediction accuracy and concentration of individual FA are expected to affect the estimates for the indices. Estimated heritabilities for the sum of *trans* FA (TRANS) were lower than for the sum of *cis* FA (CIS), and this was also reflected in the individual FA.

In the literature, estimated heritabilities for bovine milk FA composition vary largely among studies depending on sample size, breed, and method. Our estimates were generally lower than those from other studies in which FA concentrations were predicted with mid-infrared spectroscopy [5, 7, 8, 10], but they were in the same range as in the study of Krag et al. [9] in which GC was used. Our observation that individual saturated FA have higher heritabilities than unsaturated FA has been previously reported by several authors [5, 7, 9], whereas estimated heritabilities for groups of FA varied among studies. Whereas many studies support the general pattern of higher heritabilities for saturated FA than for unsaturated FA [5, 6, 8, 10], the highest estimates were found for MUFA in the study of Krag et al. [9], and for PUFA in the current study. The disparity in these results most likely reflects differences in concentrations and prediction accuracies of the FA included in the different FA groups.

### Genome-wide association studies

Phenotypic records for the 29 traits considered to be predictable, together with pre-existing records for urea and NEFA, were tested for their association with ~17,000 genome-wide distributed SNPs using a single-marker association model. We detected 200 significant marker-trait associations and the most significant associations were clustered on BTA1, 13 and 15. These QTL are further discussed below and compared with findings from other studies. All significant results are in Additional file 3: Table S3.

#### BTA13

In our study, the most relevant QTL were detected between 55.4 and 66.1 Mb on BTA13. These QTL affected the content in all short- and medium-chained, saturated de novo synthesized milk FA (i.e.; C4:0–C14:0 and DNS). Among these, the highest LRT was detected between SNP rs29018443 and C8:0 (LRT = 26.98), and this same SNP was also highly associated with C6:0, C10:0, C12:0, C14:0 and DNS. A strong candidate gene, *acyl*-*CoA synthetase short*-*chain family member 2* (*ACSS2*), lies nearby this SNP and encodes an enzyme that catalyzes the activation of acetate for de novo synthesis of short-chained FA [36]. *ACSS2* was also suggested as a candidate gene that affects de novo synthesized FA (C6:0, C8:0 and C10:0) in Dutch Holstein–Friesian [37] and Danish Jersey cattle [38], and several C16 and C18 FA in Chinese Holstein [39].

#### BTA1

In our study, the most significant association (LRT = 33.94) was between SNP rs29019625 located at 144.4 Mb on BTA1 and C18:2*cis*-9,*cis*-12. This SNP was also significantly associated to N6, C18:1*trans*-11, C15:0 and PUFA. The QTL region spanned the ~126.3–144.4 Mb region and included also significant associations to C6:0–C12:0, DNS and DHA. SNP rs29019625 lies approximately 20 kb away from the *SLC37A1* gene, which encodes a membrane bound protein that is involved in the translocation of glycerol-3-phosphate into the endoplasmic reticulum [40]. Other positional candidate genes are *ABCG1* and *AGPAT3.* The former is located at 144 Mb and is involved in macrophage cholesterol and phospholipid transport and may regulate cellular lipid homeostasis in other cell types (e.g., [41]), while *AGPAT3* is located at 146.7 Mb and encodes an acyltransferase that has a role in the de novo phospholipid biosynthetic pathway [42].

A connection between BTA1 and predominantly long-chained FA was reported in several studies. Schennink et al. [43] observed significant associations between markers on BTA1 and C18:0, C18-index and CLA-index at ~125 cM (which corresponds roughly to ~140 Mb according to their map published in Schopen et al. [44]). Bouwman et al. [37] reported a QTL region for C14:0 that is located between ~121 and 130 Mb and for C16:1 between ~146 and 161 Mb in the Dutch Holstein–Friesian population. Li et al. [39] detected significant associations with markers on BTA1 for C10:0 and C12:0 at 132 Mb and for C18:0 and C18 index at 146 Mb in Chinese Holstein. Furthermore, Li et al. [45] reported associations between BTA1 and C18 index at 142.2 Mb in Chinese Holstein and C18:0 at 146.3 Mb in a joint analysis of Chinese and Danish Holstein.

#### BTA15

The QTL region that was detected on BTA15 (between 22.6 and 29.0 Mb) affects C8:0–C14:0, DNS, C18:0, C18:1*cis*-9, CIS, *trans:cis* ratio, iodine value and total fat yield, with the highest LRT being for DNS (LRT = 25.8). This QTL is situated close to the genes encoding the following apolipoproteins APOA1, APOA3, APOA4 and APOA5 at 27.9 Mb. This QTL region is frequently cited in the literature. Bouwman et al. [37] detected associations between QTL in the region that lies from 20.5 to 27 Mb on BTA15 and two de novo synthesized FA (C10:0 and C14:0) in Dutch Holstein–Friesian. Within the same region, associations to C18:0 and C18 index in Chinese Holstein [39] and to C12:0, C14:0, and C18:1*cis*-9 in Danish Jersey [38] were reported. Furthermore, Li et al. [45] reported associations to C18:0 and C18 index at position 28.6 Mb in Chinese Holstein and at 27.3–32.8 Mb in a joint analysis of Chinese and Danish Holstein.

GWAS studies frequently report strong associations between milk FA and the genes *diacylglycerol acyltransferase 1* (*DGAT1*) on BTA14 and *stearoyl*-*coenzyme A desaturase 1* (*SCD*) on BTA26. *DGAT1* encodes an enzyme that catalyzes the final stage of triacylglycerol synthesis (e.g. [46]), while *SCD* is involved in the synthesis of monounsaturated FA by introducing a double bond in the delta-9 position of C14:0, C16:0 and C18:0, primarily [47]. No significant associations in the vicinity of *DGAT1* were detected in our study. Subsequent re-sequencing of 147 NR animals showed that they were all homozygous for the *A* variant of the *DGAT1* K232A polymorphism (not shown). In contrast to the *A* variant, the *K* variant is associated with increased fat yield, fat percentage and protein percentage and decreased milk yield and protein yield. Selection may have favored the *A* variant in the NR population, because most selection pressure was put on milk and protein yield in the breeding goal. In contrast, both allele variants of an important *SCD1* polymorphism (A293V) were found to be relatively common in the sequenced NR individuals with a MAF of 0.25 (data not shown); however a follow-up study that examined the *SCD1* region by including additional SNPs did not detect any significant associations near *SCD1* (unpublished results).

### Fine-mapping using a high-density SNP dataset on BTA13

**Fig. 1:**
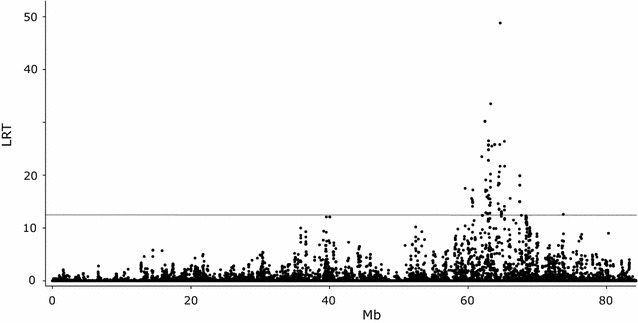
Association analysis of FA synthesized de novo (DNS) for SNPs on BTA13 from the BovineHD BeadChip. The ordinate denotes the LRT, while the abscissa denotes SNP positions in Mb. The *grey line* indicates the significance threshold (LRT = 12.12)

Subsequent analyses were performed to fine-map the BTA13 QTL that affects de novo synthesized FA and to identify potential causal variations. We began by reanalyzing the associations between all the high-density SNPs on BTA13 (n = 16,567) and the traits that were significant in the initial GWAS (i.e. C4:0–C14:0 and DNS). Somewhat surprisingly, this analysis did not point towards the prime candidate gene *ACSS2* as the most likely position of the QTL, but to a nearby gene i.e. *nuclear receptor coactivator 6* (*NCOA6*) that encodes a transcriptional co-activator, which interacts with nuclear hormone receptors. The most significant result was found for SNP rs41700740 at 64,650,276 bp which is a synonymous variant located within this gene. The LRT for this SNP ranged from 62.6 for C8:0 to 24.5 for C14:0. Significant LRT were found for ~500 SNP/trait combinations in the QTL region. As an example, results for DNS are in Fig. 1, while LRT for all SNP/trait combinations are in Additional file 4: Table S4.

### Fine-mapping using SNPs in the *NCOA6* and *ACSS2* genes at the sequence level

**Fig. 2:**
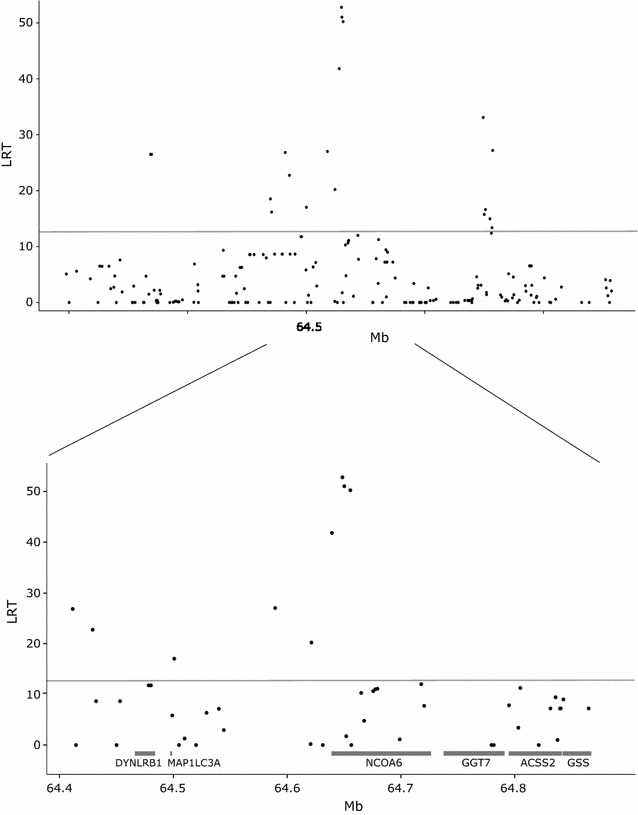
Association analysis of FA synthesized de novo (DNS) in the candidate gene region. *Top* results for the entire candidate gene region. The ordinate denotes the LRT, while the abscissa denotes SNP positions in bp. *Bottom* zoom on the region between 64.4 and 64.9 Mb. The positions of the genes in the region are indicated with *grey boxes*. The *grey line* indicates the significance threshold (LRT = 12.12)

Since our analyses pointed towards *NCOA6* and not *ACSS2* as the most likely positional candidate gene underlying the QTL, both genes were investigated in more detail. A dataset consisting of 15 SNPs within *NCOA6* and nine SNPs within *ACSS2* as well as 180 SNPs in the regions surrounding these genes was constructed by combining sequence-level polymorphisms with SNPs from the Bovine HD BeadChip. Both C6–C14 as well as DNS were reanalyzed for these SNPs using the single-SNP model. The results showed that, for C6:0–C12:0 and DNS, the highest LRT was found for SNP 99, i.e. rs41700742 at 64,648,620 bp, which is a synonymous SNP located within *NCOA6*. High LRT were also detected for SNP 100 (rs41700740 at 64,650,276 bp), SNP 102 (rs41700737 at 64,655,588 bp) and SNP 98 (rs41700745 at 64,639,392 bp). All these SNPs are localized within *NCOA6*; the former and the latter are synonymous exonic SNPs whereas rs41700737 at 64,655,588 bp is an intronic SNP. For C14:0, SNP 161 (rs43711970) at 65,246,092 bp was slightly more significant (24.2 vs. 23.8) than SNP 99. SNP 161 is located within the gene *UQCC*, which is almost 400 kb away from *NCOA6* on the telomeric side. Complete results for all traits and SNPs are in Additional file 5: Table S5. As an example, results for DNS are in Fig. 2.

In order to determine if more than one QTL segregated in the detected region, the DNS traits were re-analyzed by including the effect of SNP rs41700742 as a fixed term (not shown). The results showed that this SNP explained all the variation, which indicates that only one QTL is segregating for the DNS traits, and the signals detected for the remaining SNPs were merely due to LD between SNPs.

### Haplotype analyses

**Fig. 3:**
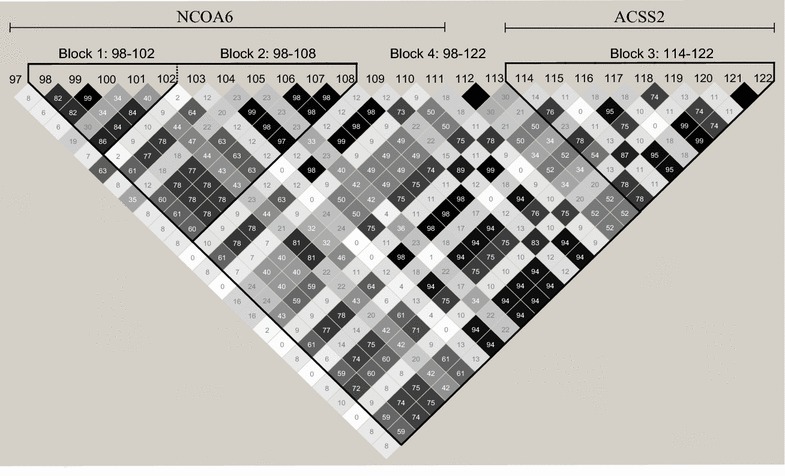
Haploview plot illustrating LD between pairwise combinations of SNPs within and between *NCOA6* and *ACSS2*. Genes are shown together with the blocks used in the haplotype analyses. *Numbers above the triangle* denote marker number in the candidate gene region. *Numbers within the triangle* are pair-wise LD between markers in the form of r^2^ * 100

Finally, to better characterize the BTA13 QTL, all the SNPs within the QTL region were grouped into haplotype blocks in order to identify the haplotypes that displayed the strongest associations to C8:0, which is a proxy for DNS. Pair-wise LD measure (r^2^) for all SNP pairs in the candidate gene region are in Fig. 3 along with four manually-constructed haplotype blocks. Within each block, each haplotype with a frequency higher than 0.01 was tested against the mean of the remaining haplotypes. Results for haplotypes with a frequency of 0.05 or more are in Table 2. The most significant effects were detected in the narrow *NCOA6* block (block 1 that included SNPs 98 to 102), which displays eight haplotypes. A frequent haplotype (denoted 1.1) was associated with higher content of short-chained FA (p = 0.00037), while haplotypes 1.2 and 1.4 were associated with lower FA content (p = 0.0000048 and 0.027, respectively). When the haplotype block was extended to include SNPs 98 to 108 in the broader *NCOA6* block (block 2, which also consisted of eight haplotypes), the differences between haplotypes were less marked. Haplotype 2.1 within this block had an identical frequency and p value as in the narrow block. The two negative haplotypes from block 1 were split into several less frequent haplotypes, with the most frequent being haplotypes 2.4 (p = 0.038) and 2.6 (p = 0.09). Block 3 covered *ACSS2* (SNPs 114 to 122) and produced even less significant results. A larger block that contained the SNPs located within both *NCOA6* and *ACSS2* (block 4, including SNPs 98 to 125 with eight haplotypes), the differences between haplotypes became more marked again. The most frequent haplotype (4.1) showed a stronger effect than the remaining haplotypes with a p value of 0.00046. In summary, the strongest associations were found for haplotypes within a rather narrow region that contained *NCOA6*. Neither the haplotypes within a larger block that included both *NCOA6* and *ACSS2* nor the block that contained only *ACSS2* were significant. Thus, the results of the haplotype analyses also suggest that *NCOA6* is a stronger positional candidate for the observed variation in de novo FA synthesis than *ACSS2*.

**Table 2:** **Haplotype analyses**

Block	Hap number	Haplotype	Effect	Freq	p value
1	1.1	GAACA	+	0.56	0.00037
	1.2	AGGCG	−	0.18	0.0000048
	1.4	AGGAG	−	0.19	0.027
2	2.1	GAACAAAGAAG	+	0.56	0.00037
	2.4	AGGCGAGGCGA	−	0.12	0.038
	2.6	AGGAGAGGCGA	−	0.09	0.09
	2.2	AGGCGAAGAAG	−	0.05	0.000000059
	2.5	AGGAGAGACGA	−	0.05	0.017
3	3.1	GACGGGGAC	+	0.62	0.125
	3.3	AAAGACGGA	−	0.12	0.043
	3.5	AAAAACGGA	−	0.08	0.032
	3.4	AGAGACAGA	−	0.06	0.08
4	4.1	GAACAAAGAAGGACAAGACGGGGACGGC	+	0.56	0.00046
	4.4	AGGCGAGGCGAGTCAAAAAGACGGAAAA	−	0.12	0.043
	4.6	AGGAGAGGCGAGTCGGAAAAACGGAAAA	−	0.08	0.034
	4.2	AGGCGAAGAAGGACAAGACGGGGACGGC	−	0.05	0.000000059

Block number, haplotype number (Hap number), haplotype, effect of haplotype (+ is higher content of C8:0 as compared to mean of remaining haplotypes in the block, − is lower C8:0 content), frequency and p-value of each haplotype. Block 1: *NCOA6*, SNPs 98 to 102. Block 2: *NCOA6*, SNPs 98 to 108. Block 3: *ACSS2*, SNPs 114 to 122. Block 4: *NCOA6* and *ACSS2*, SNPs 98 to 125

#### **NCOA6**

*NCOA6*, or *nuclear receptor coactivator 6*, encodes an essential, non-redundant multifunctional coactivator for nuclear hormone receptors and certain other transcription factors [48]. The gene is expressed in a variety of tissues, such as testis, brain, ovary, liver, fat and heart [48] and also in the mammary gland [49]. *NCOA6* is essential for embryonic development [50], it is involved in cell survival, growth, wound healing and energy metabolism [51], and is important for normal mammary gland development [52]. Different *NCOA6* isoforms are expressed in the mouse mammary gland at different developmental stages including adult virgin, pregnancy, lactation and involution [48].

To the best of our knowledge, no studies have specifically investigated the role of *NCOA6* in milk fat synthesis. However *NCOA6* is known to be a ligand for transcription factors such as PPARα and PPARγ [53], and thus, its effect could be through these. PPARγ affects expression of genes that are involved in fatty acid transport such as *LPL*, *CD36* and *ACSL1* [54], and is proposed as a major regulator of bovine milk fat synthesis [2]. In a study on the gene regulatory networks in lactation, *NCOA6* (in that study denoted *PRIP*) was identified as one of the factors involved in PPARα/RXRα signaling [55]. Therefore, *NCOA6* could be a functional as well as a positional candidate for the QTL on BTA13.

Our study did not identify any candidate causal polymorphisms underlying the QTL. The three SNPs with the highest LRT are either synonymous or intronic and therefore do not directly alter the protein sequence. However, introns can harbor important regulating elements such as binding sites for transcription factors and sites that affect alternative splicing. Synonymous SNPs are also suggested to have important biological roles, as they may have an impact on critical cis-regulating sequences, alter mRNA structure and influence translational speed [56]. Further analyses will be undertaken in order to investigate the nature of the QTL on BTA13 and other QTL that have an effect on bovine milk FA composition.

## Conclusions

Using a combined dataset of high-resolution genotypes and FTIR phenotypes, our GWAS detected significant QTL for milk fatty acids on BTA1, 13 and 15. On BTA13, the QTL for de novo fatty acid synthesis mapped close to a known candidate gene (*ACSS2*), but subsequent refined analyses highlighted that *ACSS2* had little effect and that SNPs within the nearby *NCOA6* gene were responsible for the observed QTL. To date, the functional role of *NCOA6* in milk fatty acid synthesis is unclear, but one possible effect could be that it is a ligand for the transcription factor PPARγ, which is suggested to be a major regulator of milk fat synthesis.

## Additional files

**Additional file 1: Table S1.** SNPs genotyped for the candidate gene map, with rs numbers, positions in base pairs and primer sequences.

**Additional file 2: Table S2.** Results for calibration of FTIR-spectra against GC-FID reference data, correlations between each FA and total fat percentage, and estimates of variance components with standard errors.

**Additional file 3: Table S3.** GWAS results.

**Additional file 4: Table S4.** Results from single-marker association analyses on BTA13 data from the BovineHD BeadChip.

**Additional file 5: Table S5.** Results from single-marker association analyses on SNPs in the candidate gene region.
